# Role of servicescape in patients’ clinic care waiting experience: Evidence from developing countries

**DOI:** 10.1371/journal.pone.0311542

**Published:** 2024-10-15

**Authors:** Abdul Rauf, Norhilmi Muhammad, Hamid Mahmood, Yuen Yee Yen, Muhammad Haroon Rashid, Warda Naseem

**Affiliations:** 1 Faculty of Business and Management, Universiti Sultan Zainal Abidin, Kuala Terengganu, Malaysia; 2 Faculty of General Studies and Advanced Education, Universiti Sultan Zainal Abidin, Kuala Terengganu, Malaysia; 3 Department of Management Science, TIMES Institute, Multan, Pakistan; 4 Faculty of Business, Multimedia University, Melaka, Malaysia; 5 School of Economics and Management, Harbin Institute of Technology, Harbin, China; National University of Sciences and Technology, PAKISTAN

## Abstract

The aim of this research is to investigate the role of servicescape on re-patronage and recommended intention through pleasure feeling and satisfaction in healthcare settings that put substantial contribution in the process of healthcare service delivery. Data were collected through cross-sectional convenience sampling via a self-administered survey questionnaire from 431 clinical outpatients who revisit the same hospital of metropolitan areas of Punjab, Pakistan. Structural Equation Modeling (SEM) was carried out for path analysis through AMOS (24.0 V), while statistical measures were analyzed using SPSS (25.0 V). The present study results revealed that patients’ intention optimistically triggered through partial mediation and affirm the direct and indirect association with servicescape. It also revealed that patient-recommended and re-patronage intentions to visit the clinic were statistically substantial and positively influenced by intervening constructs of pleasure feeling and satisfaction. Additionally, it is found that servicescape and pleasure feeling contributed to 30% change in satisfaction. Moreover, pleasure feeling, and satisfaction contributed to 50% change in re-patronage and 31% change in recommendation intention of the patients. The current study findings contribute significantly to servicescape literature from a theatrical perspective and reevaluate the patterns and operations in healthcare. It also helps managers and administrators of private hospitals to make strategies to increase patient satisfaction.

## Introduction

Patients with different health conditions experience treatment burden from outpatient appointments. Waiting for an appointment in the clinic can generate emotions, such as anxiety and stress [1], which affect their behavioral outcomes [2]. Resega et al [3] studied patients’ emotional condition in waiting rooms and discovered that patients felt anxious; nevertheless, patients were waiting for serious treatment or routine follow-up. Patients with different health conditions generate negative emotions while waiting at the clinic, which affect their experiences and future intentions. Patient experience also plays an important role in elucidating servicescape in healthcare settings. As patients navigate complexities regarding outpatient appointment, their satisfaction and emotional well-being intensively impact patient’s healthcare journey. Furthermore, understanding patients’ emotional burdens during clinic visits and the role of servicescape in shaping patients’ perceptions are essential for healthcare providers to tailor services. Therefore, patients’ idiosyncratic insight is very important for improving their experience to design services [4]. Additionally, empirical evidence indicates that patients’ perceptions regarding the clinics are formed by the physical environment and safety [5]. This indicates that patient’s clinical care experience may be assessed through the physical dimensions of the service environment in which it is provided. Service environment is often studied using ’servicescape’ concept in clinical context [6], that relies on spatial layout, ambiance, and signage (i.e., noise, temperature, and lightning) [7], which have shown significant positive effect on patient experience and subsequent satisfaction [8, 9].

Several studies have investigated the servicescape elements (color, music, scent, and light) with customer satisfaction and behavior [10–12], but the role of pleasure feelings on patient satisfaction with servicescape in the healthcare sector needs to be addressed. Pleasure feeling is predominantly important in dispersive and complex settings (e.g., hospitals/clinics), where patients rely on nonclinical and clinical services. Unlike retail and hospitality service settings, where physical environment and pleasure feelings are highly valued [13, 14]. Although pleasure feelings during service consumption process are less important than functional aspects in utilitarian services (e.g., banks and healthcare services), but their effective response plays an imperative role in patient satisfaction [15, 16]. Manzoor et al [17] defined patient satisfaction as “feelings of happiness, fulfillment, and pleasure towards service provider and its services.” Satisfaction is driven from patient/customer expectations with service providers and tempered by individuals’ emotions toward environmental surroundings [18]. Satisfaction is also considered as more valuable and reliable determinant for maintaining patient’s long-term behavior [19]. However, healthcare service providers who overlook the importance of customer satisfaction may risk losing patients [20]. In other words, higher patient satisfaction leads to greater patient retention [21, 22], and willingness to recommend [23, 24]. Moreover, role of recommended intention and re-patronage intention is well established in hedonic service context, but their impact in utilitarian services, particularly in healthcare, has not been sufficiently investigated [25].

**Fig 1:**
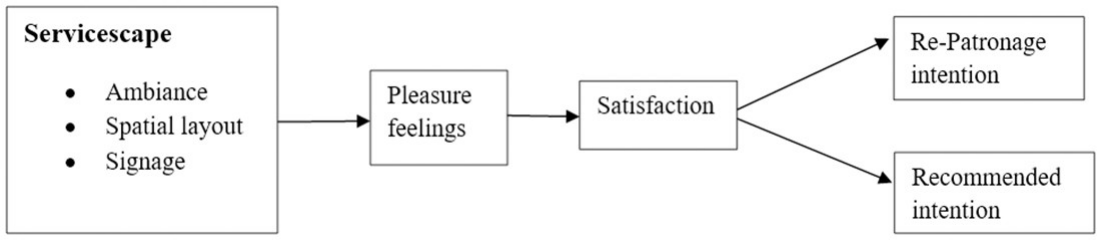
Research framework.

The current study was conducted in a metropolitan city in Punjab, Pakistan, due to its significant population size of 53% and substantial proportion of hospitals. The current research also aimed to investigate the effect of pleasure feelings and satisfaction on re-patronage intention and recommended intention with servicescape in healthcare service settings. Additionally, it explores the mediational effect of pleasure feelings between satisfaction and servicescape (Fig 1). The results of present study contribute to the body of knowledge and help the administrators and managers to improve servicescape and increase patient satisfaction in a service environment. More precisely, better understanding of user satisfaction can guide service investment decisions and facilitate the creation of patient-focused hospitable environment. It will also help hospital managers with decision-making points to build strong environment that can attract and retain patients in this intensive healthcare competition.

## Literature review

### Servicescape and pleasure feeling

The concept of servicescape, which denotes the physical environment of a service platform, was initially introduced by Bitner [7]. It is denoted with term "atmospheric" which was created by Kotler [26], highlighting it as an effort to design the buying environment to produce buyer-specific emotional effects, that enhance their purchase probability. Servicescape environmental stimuli are characterized by three facets: ambiance, spatial layout, and signage [27]. The first facet is ambiance, which refers to intangible characteristics that stimulate human preceptory and influence patients’ response to the physical environment through cleanliness, visuals, and ambient conditions [28]. The second facet is the spatial layout, which refers to the settings between objects, such as machines, equipment, or furniture in a service area. The third facet is signage, which denotes physical signals to the patients and communicates general meanings during the service process [29]. Signage creates an organizational image, influences patient needs, and improves the service environment [30]. All of these facets [ambiance, spatial layout, and signage] generate attitudes about how individuals behave and generate pleasure feelings among them [8]. Pleasure is considered as significant factor in shaping environmental experiences and evokes people to engage in a behavioral approach [31]. Additionally, patients tend to feel dominant when servicescape is perceived as flexible, ordered, and rated more intensely associated with patients’ submissive feelings. It also intends to create certain emotions that generate negative or positive feelings among patients [32]. Thus, we propose:

H1: Servicescape positively relates to pleasure feelings.

### Pleasure feeling and satisfaction

Pleasure feelings are described as hedonistic emotions, please, and the degree to which an individual feels happy [33]. Pleasure feelings also play an important role in patient response during service experience [34]. Positive emotions with service experience trigger a feeling of pleasure, which acts as a driver of satisfaction [35]. Patient satisfaction with the service environment results in perceptions and specific emotions [36]. Therefore, this study argues that positive emotions express a better service environment and indicate higher patient satisfaction. Additionally, past studies have examined the relationship of pleasure feelings with satisfaction in tourism and retail services [37]. Despite prior studies, there has been minimal effort to determine the relationship between pleasure feeling and patient satisfaction in healthcare services settings. Therefore, we propose the following hypotheses:

H2: Pleasure feelings positively relate to satisfaction.

### Satisfaction and re-patronage, recommended intention

Satisfaction is an adequate sensation of comfort and joy, resulting in fulfillment of the cognitive evaluation of experiences, events, and thoughts [37]. Patient satisfaction refers to certain service delivery characteristics, including interpersonal communication and physical environment [38], that summarize satisfaction’s effective and cognitive components. Although few studies have investigated the relationship between patient satisfaction with recommended intention and re-patronage intention in healthcare, despite that extensive literature argues that intention is an imperative outcome of satisfaction [39]. Intentions refer to idiosyncratic judgment about patient’s future behavior that normally functions as an outcome variable [40]. Hellier et al. [41] defined re-patronage intention as an individual’s judgment regarding buying again from the same service provider, considering his/her current situation and circumstances. It also refers to the patient’s intention to recommend services to others [25], and intends to raise the scope and scale of the relationship [42]. Additionally, satisfied patients in the service environment intend to revisit the same hospital and recommend it to others [43]. Therefore, in the present study, re-patronage intention and recommended intention are theorized as patients’ inclination to visit same hospital and recommend it to others for different medical purposes.

H3: Satisfaction positively relates to re-patronage intention.
H4: Satisfaction positively relates to recommended intention.

### Pleasure feeling as mediator

Pleasure feeling is a potential mediator because it is known to change human emotions and hold a state of deep interest. Generally, people react emotionally before assessing a situation cognitively; nevertheless, cognition may come first because of the ability of human mind to plan and imagine the future [44]. Therefore, cognition and thoughts release emotions that influence patient satisfaction [45], and link patient’s positive or negative emotions with the healthcare service environment. Additionally, Batra and Tanej [46], found that servicescape significantly affects patients’ emotions and cognition in the service environment. Drawing on these theoretical arguments, the present study postulates the following hypothesis.

H5: Pleasure feelings mediate the relationship between servicescape and satisfaction.

### Satisfaction as mediator

Patient satisfaction is a reaction or decision that follows cognition and emotions [47]. This reaction refers to a specific concentration [physical environment] determined in a particular phase [48]. Therefore, Ge et al. [49] argued that satisfaction is the extent of pleasure felt by patients, which results from their ability to fulfill expectations and desires. On the other hand, Ruiz Fernandez et al. [50], expressed that satisfaction comes from positive perceptions and emotions developed by customers or can be measured by the degree of pleasure. This suggests that when patients feel pleasure from the service environment, it may influence their satisfaction. Furthermore, Hossain et al. [51], stated that the behavioral intention of the customer is regarded as an extension of satisfaction. When patients feel pleasure, they will probably be satisfied with the environment, leading to re-patronage and recommended intention. Previous studies also prove that satisfaction has a direct effect on re-patronage intention [52], and recommended intention [53]. Thus, we propose.

H6: Satisfaction mediates the relationship between pleasure feelings and re-patronage intention.
H7: Satisfaction mediates the relationship between pleasure feelings and recommended intention.

## Methods

The current study followed positivist paradigm to achieve study objectives and investigate causal relationship among constructs. According to Creswell [54] and Rasheed et al [55] the quantitative research design determines the conceptual model investigation. Therefore, researcher followed the quasi-experimental quantitative research design that consists of diverse and multiple data collection measures including close & open-ended survey, secondary data, and observations.

### Target population, sampling, and sample size

The current study targets patients aged between 20 and 41 or older, as this age range allows comprehensive data collection across various demographic groups. The selection of a hospital increasingly reflects public identity, encompassing values and beliefs. Consequently, outpatients are chosen as a sample to understand patients’ perspectives on global sustainable healthcare settings. The target population of present research was the outpatients of metropolitan areas of Punjab, Pakistan. Patients were selected as a sample because they have firsthand experience with healthcare services and are aware of healthcare-related issues.

A non-probability convenience sampling technique was used to collect data from outpatients due to its time-saving and ease of applicability nature. The current study also used vigorously incorporating statistical methods to enhance convenience sampling procedure and eliminate biasness. This method regulates potential biases and demographics for data weightage, ultimately advancing the assurance of study findings and strengthening the convenience sampling technique’s reliability [56]. According to Roscoe [57] the minimum sample of 30 and maximum sample size of 500 are considered appropriate for behavioral studies. The sample greater than 200 offer suitable margin error and contrary to this, 200 sample size is required for Structural Equation Modelling (SEM) [58, 59]. So, considering the potential biasness, the sample size of present research is 512.

### Instrument

The current research model, based on servicescape, pleasure feeling, and satisfaction, was hypothesized to envisage patients’ recommendations and re-patronage intention. An adapted instrument was developed from extant studies to validate the scale of this study. A four-item scale, focusing on ambient conditions, spatial layout, and signage, was adapted from Sag et al. [60], and Akmaz and Cadiric [61]. Pleasure feeling was measured using four items adapted from Kim and Moon [62]. The seven satisfaction items were adapted from Larsen et al. [63]. Simultaneously, five and three items were adapted to measure re-patronage intention Abbasi et al. [64], and recommended intention Al-Ansi et al. [23] respectively. All these constructs were measured with five-point Likert scale.

### Data collection, time frame and response rate

This cross-sectional research uses numerous statistical methods to examine the contextual approach. The present study comprises patients’ clinical care waiting experience support to encounter the wayfinding waiting room environment to advance healthier consideration regarding contextual issues. This study used a survey method (pencil-paper based) for data collection from outpatients via convenience sampling. The data were collected between august 2023 and September 2023 with the help of seven research scholars. For ethical considerations, researcher affirmed that participants’ responses used for academic purposes, and their identities remained anonymous. After obtaining consent, 512 questionnaires were distributed and only 476 responses were received. Out of 476 responses, 431 were finalized after eliminating missing information that availed an 84.4% response rate. The dominant respondents were male (57.1%, n = 246) and belonged to the age group of 41 or above (42.9%, n = 185). The demographic profiles are highlighted in detail in Table 1.

**Table 1:** Respondents’ profile (n = 431).

Variables	Frequency	Percentage
Gender		
Male	246	57.1%
Female	185	42.9%
Age		
20 & Below	22	5.1%
21–30	80	18.6%
31–40	144	33.4%
41 or above	185	42.9%
Education		
High School	129	29.9%
Some College	175	40.6%
Postgraduate	119	27.4%
PhD	08	1.9%
Residence		
Urban	237	55.2%
Rural	194	44.8%
Income [Monthly]		
Below Rs. 40,000	44	10.2%
Rs. 41000–60,000	93	21.6%
Rs. 61000–80,000	188	43.6%
81000 and above	106	24.6%
Switching Experience		
Yes	202	46.9%
No	229	53.1%

Source: Based on calculation of SPSS software

### Common method bias

While considering data screening, the present study found that not a single item was missed from the required information. According to Kline [65], the rule of thumb for skewness ranges from −1 to + 1 for every construct, confirming the validity of the present study data. Respondents were informed about the purpose and constructs of the present study to lessen the common method influence. The predictor and criterion constructs’ data were collected cross-sectionally, contradicting the assumptions of [66]. This study applied Harman’s single-factor test to check common method bias [67]. The results revealed that one factor elucidated less than 53% of variance and guaranteed the nonappearance of common method bias.

### Data analysis method

The present study applied structural equation modeling (SEM) to establish the predictive association between study constructs using AMOS (24.0). The model was developed based on path analysis to identify the direct and indirect associations between servicescape and recommended and re-purchase intentions. SEM was used in the current study due to the relatively large dataset; in contrast, PLS-SEM is recommended in complex models. Furthermore, the present study focused on assessing the association among outcomes of predictive constructs, and SEM is considered more appropriate for predictive modeling [68, 69].

## Results

### Demographic of respondents

Respondents’ demographics are presented in Table 1. Out of the 431 respondents, 246 (57.1%) were male and remaining 185 (42.9%) were female. The majority of the respondents were aged 41 or above with a frequency of 185 (42.9%). In the qualification section, most respondents reported having college education, with the frequency of 175 (40.6%). The majority, 237 (55.2%) of the respondents were from urban areas and the remaining 194 (44.8%) were from rural areas. The next section illustrates the income of the respondents, in which income between Rs. 61,000 to 80,000 had highest frequency of 175 with percentage of 40.6%. The last section referred to the switching experience of the respondents, in which 229 (53.1%) of the respondents had no prior switching experience.

### Measurement model

The reliability and validity of the constructs were measured using confirmatory factor analysis (CFA). The results show assessment of the measurement model within the acceptable range of model fit (χ2/df = 2.298, df = 196, GFI = 0.960, NFI = 0.931, RFI = 0.92, CFI = 0.95, TLI = 0.952, RMSEA = 0.055) [70]. Standardized factor loading of all items exceeded a threshold of 0.50, with p< 0.001. The average variance extracted was also greater than 0.60, the threshold level of AVE, and fell within the range of 0.657 to 0.764, as shown in Table 2. Further, composite reliability is also greater than the threshold level of 0.70 (63), and falls within the range of 0.816 to 0.921. The convergent validity of the current study supports these measures. Finally, the present study also established discriminant validity, as shown in Table 3.

**Table 2:** Factor loadings, average variance extracted and composite reliability.

Construct	Dimension	Item	FL	AVE	CR	Α
***Servicescape***	***Spatial***	A2	.81	.712	.881	.821
		A3	.87			
		A4	.85			
	***Signage***	E1	.84	.689	.816	.883
		E2	.82			
	***Ambient***	RI1	.86	.757	.862	.875
		RI4	.88			
***Pleasure Feeling***		RES1	.83	.744	.921	.894
		RES2	.86			
		RES3	.89			
		RES4	.87			
***Re-Patronage Intentions***		AA1	.81	.657	.884	.887
		AA2	.77			
		AA3	.84			
		AA4	.82			
***Satisfaction***		S3	.82	.698	.902	.865
		S4	.84			
		S5	.86			
		S6	.82			
***Recommended Intentions***		T1	.88	.764	.907	.779
		T2	.82			
		T3	.92			

**Table 3:** Discriminant validity.

**Construct**	**VIF**	**SS**	**P**	**S**	**RPI**	**RCI**
**SS**	1.411	**.85**				
**P**	1.923	.68	**.86**			
**S**	1.109	.47	.52	**.84**		
**RPI**	1.121	.45	.53	.67	**.81**	
**RCI**	1.129	.47	.47	.48	.46	**.87**

Note: SS = Servicescape, P = Pleasure, S = Satisfaction, RPI = Re-Patronage Intention, RCI = Recommended Intention

### Path analysis via CB-SEM

**Fig 2:**
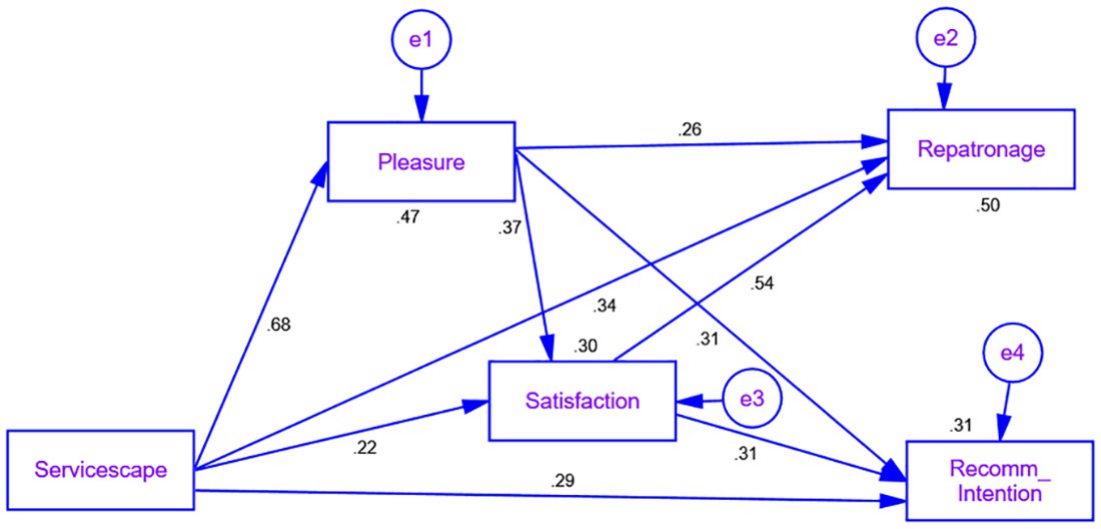
Structural equation modeling.

Multicollinearity did not exist in the model because all assessed variance inflation factor (VIF) values varied from 1.112 to 1.866 and were below the requisite threshold level of 3.0. Table 4 presents the proposed hypotheses (H1-H4). All of the assumptions confirmed that servicescape positively influences customer pleasure feeling (β = .681, p.000), secondly pleasure feeling positively affects customer satisfaction (β = .374, p.000), Thirdly, satisfaction positively influences re-patronage (β = .546, p.000), and customer satisfaction positively influences the recommended intentions of outpatients (β = .315, p.000) (shown in Fig 2).

**Table 4:** Path analysis and path coefficients.

Path Analysis			Estimate	S.E.	C.R.	P	Decision
P	←	SS	.681	.054	13.403	***	Supported
S	←	SS	.223	.069	3.313	***	Supported
S	←	P	.374	.067	5.383	***	Supported
RPI	←	P	.262	.045	4.942	***	Supported
RCI	←	P	.311	.054	5.298	***	Supported
RCI	←	S	.315	.056	5.507	***	Supported
RPI	←	S	.546	.052	9.326	***	Supported

Note: SS = Servicescape, P = Pleasure, S = Satisfaction, RPI = Re-Patronage Intention, RCI = Recommended Intention

### Mediation analysis

The current study investigates the mediational effect of using bootstrapping Maximum Likelihood Estimation (MLE) approach with 1000 bootstrap samples with 95% confidence interval. Results showed a substantial indirect effect of servicescape on satisfaction via pleasure feeling (β = .429*.363 = .155). Further, it also shows the indirect effect of pleasure feeling via satisfaction with re-patronage intention (β = .411*.463 = .191) and recommended intention (β = .392*.397 = .157). Additionally, it found that change of 47% in pleasure feeling induced by servicescape. The predictive capacity (R2) of the total variance in the dependent variable was identified as a result of changes in independent factors. Furthermore, servicescape and pleasure feeling contributed to 30% change in satisfaction. Thirdly, pleasure feeling, and satisfaction contributed to 50% change in re-patronage. Lastly, pleasure feeling, and satisfaction contributed to 31% change in recommendation intentions of the patients. The study also used Cohen’s (64) method to calculate the effect magnitude because the small, medium, and large threshold values of *f* 2 should be 0.02, 0.15, and 0.35, respectively. Hence, the present research framework proposes that pleasure feelings (*f* 2 = 0.4231) have a large effect size on mediation. Additionally, correlation (*f* 2 = 0.1934) observed between pleasure feelings and re-patronage in relationship to customer satisfaction. Lastly, satisfaction has (*f* 2 = 0.1331) a medium effect size of mediation between the relationship of pleasure feeling of customers and recommendation intentions of outpatients to visit the clinics.

## Discussion and conclusion

Numerous studies have prevailed in the literature on clinical outpatient waiting practices regarding servicescape facets [71], and stimulatingly observed with ambiance, spatial layout, and signposts that statistically influence clinical outpatients’ intentions while waiting. The present study revealed that such facets pave the way for satisfaction, eventually triggering an optimistically perceived performance that converts it into re-patronage and recommended intentions. Findings also revealed that emotional feedback and distinctiveness of waiting experience, including effective management of servicescape facets, significantly enhance overall satisfaction and intentions regarding the clinical waiting experience. The combination of ambiance, spatial layout, signage, feelings of pleasure, and satisfaction aptitude influence the clinical outpatient intention [60, 72]. Correspondingly, pleasure-based satisfaction prospectively extents superior perfection while visiting the first time, triggering the repetition of clinical outpatients.

Additionally, pleasant experiences wielded a considerable and inclusively optimistic impact on other aspects alleged more enthusiastically [73]. The present study’s findings are also consistent with an extant study because experiential constructs, re-patronage, and recommended intentions played a considerable role through repeated visits, emotive attachment, and personal recommendation for the sake of instantaneous precursors [25]. Consequently, re-patronage and recommended intentions primarily rely on clinical outpatient satisfaction in healthcare settings [48, 74]. The findings revealed that surveillance activities, i.e., checking-in and waiting, give an optimistic impression for enhancing pleasure feelings that shape potential satisfaction. Prominently, facets of servicescape experience positively contribute toward pleasure feeling that causes outpatient satisfaction.

### Theoretical implications

The past studies have investigated the role of servicescape in marketing literature and environmental psychology, but very few studies have examined the servicescape with pleasure feeling in healthcare sector. Therefore, this study contributes to relationship among servicescape, pleasure feeling, and satisfaction with recommended intention, re-patronage intention, and enhance the knowledge in the field of healthcare that remains under investigated. This study fills the literature gap a) by investigating servicescape impact on patient behavioral intention and b) by exploring the mediating effect of pleasure feeling between servicescape and satisfaction. The current study also contributes to the theoretical advancement by highlighting the role of servicescape in enhancing patients pleasure feeling in waiting areas. Additionally, current study theoretically validates the servicescape facets within the healthcare setting which require substantial investment. These insights provide understanding of how servicescape impacts in patient behavioral intention particularly pleasure feeling and satisfaction which navigate the positive experience and foster the re-patronage intention and recommended intention of the patient.

### Practical implications

It is very important for study discussion to comprehensively interpret practical insights for the health service managers and professionals. The present study findings offer significant effort to reduce disparities and provide evidence-based support in decision making process. It also revealed that healthcare management should introduce telemedicine services, mobile health clinics, and local practitioners’ involvement. Additionally, practitioners and healthcare management should initiate tailored training programs to increase patient engagement, promote cultural aptitude, establish gender-sensitive healthcare, and patient compliance among rural and urban settings. Lastly, recruitment of skilled healthcare professionals may increase patients revisit intention and improve hospital performance.

### Limitations and future direction

The present study has some limitations that offer opportunities for potential researchers. The first and foremost limitation is the sample size, which is relatively small, and the current study is based on cross-sectional data. In the future, researchers must use a longitudinal study design that might create diverse expectations for clinical outpatients. The data collection procedure is quite difficult because the study targets only outpatients actively found in clinics; in the future, it can also add some hospitals for more generalizability. Pakistan is a developing country, so the financial condition of outpatients and the stress of associated illness undeniably constrained the outpatient’s willing participation in this survey to encounter this situation, clinic staff gave some assistance, but unfortunately, they were not a part of this survey. For future research, potential researchers should set a large sample size in the same or diverse healthcare settings to authenticate the present study findings. Further research could also include staff members and user groups with diverse healthcare settings to identify the effects on their waiting experiences. Additionally, the present research relied on a quantitative approach, and in the future, qualitative or mixed methods might be used to extend outpatients’ experiences in forthcoming circumstances.

## Supporting information

S1 Dataset The data set used in this article for discussion and analysis. (XLSX)

S1 Questionnaire The data collection tool. (DOCX)
