# Supplementary material for: Role of servicescape in patients’ clinic care waiting experience: Evidence from developing countries
Source: PLoS One. 2024 Oct 15;19(10):e0311542. doi: 10.1371/journal.pone.0311542 (PMC11478909; doi:10.1371/journal.pone.0311542)
Supplement: S1 Questionnaire — (DOCX) [file pone.0311542.s002.docx]

**Role of servicescape in patients' clinic care waiting experience among developing countries**

The aim of the study is to investigate how servicescape effect on patient’s behavioral intention. It also explores factors that may strengthen or weaken this relationship.

Dear respondent, kindly fill up this information and return. Any information obtain for this purpose will be kept strictly confidential and will only be used for academic purpose. Your cooperation will be highly appreciated in this regard.

**SECTION A: DEMOGRAPHIC**

Please fill in the blank or tick (/) the appropriate response.

What is your Gender?

1. Male B) Female

Which age group do you belong to?

1. 20 & Below C) 31-40
2. 21-30 D) 41 or above

What is your education?

1. High school D) Postgraduate
2. Some college E) PhD

Residence

A) Urban B) Rural

Monthly Income?

1. Below Rs. 40,000 C) Rs. 61,000 to 80,000
2. Rs. 41,000 to 60,000 D) Rs. 81,000 and above

Switching Experience

A) Yes B) No

**Section B**

|  | **Servicescape** | 1 | 2 | 3 | 4 | 5 |
| --- | --- | --- | --- | --- | --- | --- |
|  | **Ambient Condition** |  |  |  |  |  |
| 1 | Hospital temperature was satisfying. |  |  |  |  |  |
| 2 | Air-conditioning wasn’t disturbing while waiting in hospital. |  |  |  |  |  |
| 3 | The noise wasn’t disturbing in hospital. |  |  |  |  |  |
| 4 | The smell was not unsatisfying in hospital. |  |  |  |  |  |
|  | **Spatial lay out** |  |  |  |  |  |
| 1 | It was easy to enter the hospital from the parking lot. |  |  |  |  |  |
| 2 | The hospital equipment worked properly. |  |  |  |  |  |
| 3 | The hospitals’ decorations were pleasing. |  |  |  |  |  |
| 4 | I could easily park my car in hospital parking. |  |  |  |  |  |
|  | **Signage** |  |  |  |  |  |
| 1 | The signs in the hospital were adequate. |  |  |  |  |  |
| 2 | I could easily see the signs in the hospital. |  |  |  |  |  |
| 3 | I could easily understand the signs in the hospital. |  |  |  |  |  |
| 4 | Because of the signs, I could easily find my way around the hospital. |  |  |  |  |  |

|  | **Pleasure Feelings** | 1 | 2 | 3 | 4 | 5 |
| --- | --- | --- | --- | --- | --- | --- |
| 1 | The overall feeling I get from this hospital was unhappy. |  |  |  |  |  |
| 2 | The overall feeling I get from this hospital was delighted. |  |  |  |  |  |
| 3 | The overall feeling I get from this hospital was annoyed. |  |  |  |  |  |
| 4 | The overall feeling I get from this hospital was bored. |  |  |  |  |  |

| **Satisfaction** | | **1** | **2** | **3** | **4** | **5** |
| --- | --- | --- | --- | --- | --- | --- |
| 1 | How would you rate the quality of hospital service you received? |  |  |  |  |  |
| 2 | Do you get the kind of service you want from hospital? |  |  |  |  |  |
| 3 | To what extent, hospital has met your needs? |  |  |  |  |  |
| 4 | If a friend were in need of similar help, would you recommend hospital to him/her? |  |  |  |  |  |
| 5 | How satisfied are you with the amount of help you received from hospital? |  |  |  |  |  |
| 6 | In an overall general sense, how satisfied are you with the service you received from hospital? |  |  |  |  |  |
| 7 | If you were to seek healthcare treatment again, would you come back to same hospital? |  |  |  |  |  |

| **Re-patronage intention** | | **1** | **2** | **3** | **4** | **5** |
| --- | --- | --- | --- | --- | --- | --- |
| 1 | I would like to revisit hospital when need healthcare. |  |  |  |  |  |
| 2 | I will return to the hospital of my choice to get the next health care. |  |  |  |  |  |
| 3 | I will recommend the hospital of my choice to the family as a health care choice. |  |  |  |  |  |
| 4 | Taking my past experience with this hospital into account affects my willingness to visit this hospital again. |  |  |  |  |  |
| 5 | This hospital would be my first choice over other hospitals. |  |  |  |  |  |

|  | **Recommended Intention** | 1 | 2 | 3 | 4 | 5 |
| --- | --- | --- | --- | --- | --- | --- |
| 1 | I will recommend this hospital to others. |  |  |  |  |  |
| 2 | I will say positive things about this hospital to others. |  |  |  |  |  |
| 3 | I will encourage friends and relatives to visit this hospital. |  |  |  |  |  |

***Thank you…..***
